# Bis(2,6-dihy­droxy­benzoato-κ^2^ *O* ^1^,*O* ^1′^)(nitrato-κ^2^ *O*,*O*′)bis­(1,10-phenanthroline-κ^2^ *N*,*N*′)dysprosium(III)

**DOI:** 10.1107/S1600536810048348

**Published:** 2010-11-24

**Authors:** Weiqin Dong, Yaling Cai, Bo Hong, Hongxiao Jin

**Affiliations:** a College of Materials Science and Engineering, China Jiliang University, Hangzhou 310018, People’s Republic of China

## Abstract

In the mononuclear title complex, [Dy(C_7_H_5_O_4_)_2_(NO_3_)(C_12_H_8_N_2_)_2_], the Dy^III^ atom is in a distorted bicapped square-anti­prismatic geometry formed by four N atoms from two chelating 1,10-phenanthroline (phen) ligands, four O atoms from two 2,6-dihy­droxy­benzoate (DHB) ligands and two O atoms from a nitrate anion. Inter­molecular π–π stacking inter­actions between the phen and DHB ligands [centroid–centroid distances = 3.542 (4) and 3.879 (4) Å] and between the pyridine and benzene rings of adjacent phen ligands [centroid–centroid distance = 3.751 (4) Å] stabilize the crystal structure. Intra­molecular O–H⋯O hydrogen bonds are observed in the DHB ligands.

## Related literature

For a related structure, see: Zheng *et al.* (2010 ▶).
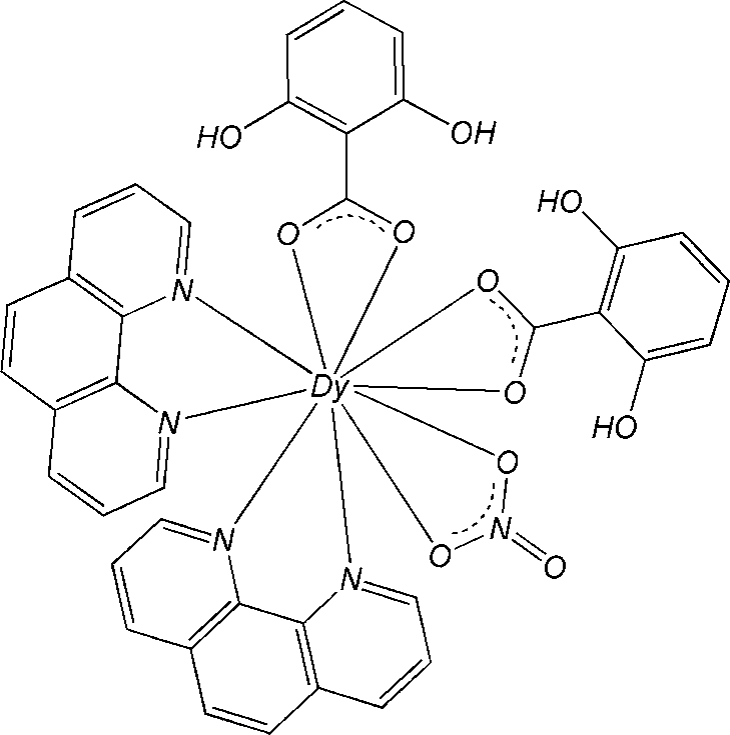


## Experimental

### 

#### Crystal data

[Dy(C_7_H_5_O_4_)_2_(NO_3_)(C_12_H_8_N_2_)_2_]
*M* *_r_* = 891.14
Monoclinic, 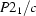

*a* = 11.1268 (3) Å
*b* = 26.7587 (4) Å
*c* = 14.2703 (5) Å
β = 127.280 (2)°
*V* = 3380.72 (18) Å^3^
*Z* = 4
Cu *K*α radiation
μ = 12.46 mm^−1^
*T* = 298 K
0.35 × 0.32 × 0.30 mm

#### Data collection

Oxford Diffraction Gemini S Ultra CCD diffractometer
Absorption correction: multi-scan (*CrysAlis PRO*; Oxford Diffraction, 2006 ▶) *T*_min_ = 0.097, *T*_max_ = 0.118
11595 measured reflections
5923 independent reflections
4674 reflections with *I* > 2σ(*I*)
*R*_int_ = 0.043

#### Refinement

*R*[*F* ^2^ > 2σ(*F* ^2^)] = 0.051
*wR*(*F* ^2^) = 0.137
*S* = 1.00
5923 reflections
496 parameters
H-atom parameters constrained
Δρ_max_ = 1.68 e Å^−3^
Δρ_min_ = −1.16 e Å^−3^

### 

Data collection: *CrysAlis PRO* (Oxford Diffraction, 2006 ▶); cell refinement: *CrysAlis PRO*; data reduction: *CrysAlis PRO*; program(s) used to solve structure: *SHELXS97* (Sheldrick, 2008 ▶); program(s) used to refine structure: *SHELXL97* (Sheldrick, 2008 ▶); molecular graphics: *DIAMOND* (Brandenburg & Berndt, 1999 ▶); software used to prepare material for publication: *SHELXL97*.

## Supplementary Material

Crystal structure: contains datablocks I, global. DOI: 10.1107/S1600536810048348/hy2381sup1.cif

Structure factors: contains datablocks I. DOI: 10.1107/S1600536810048348/hy2381Isup2.hkl

Additional supplementary materials:  crystallographic information; 3D view; checkCIF report

## Figures and Tables

**Table 1:** Selected bond lengths (Å)

Dy1—N1	2.597 (5)
Dy1—N2	2.556 (4)
Dy1—N3	2.545 (5)
Dy1—N4	2.515 (5)
Dy1—O1	2.442 (4)
Dy1—O2	2.557 (4)
Dy1—O5	2.458 (4)
Dy1—O6	2.444 (5)
Dy1—O9	2.604 (5)
Dy1—O10	2.482 (5)

**Table 2:** Hydrogen-bond geometry (Å, °)

*D*—H⋯*A*	*D*—H	H⋯*A*	*D*⋯*A*	*D*—H⋯*A*
O3—H27⋯O1	0.82	1.86	2.577 (7)	146
O4—H31⋯O2	0.82	1.86	2.588 (6)	148
O7—H34⋯O5	0.82	1.91	2.592 (7)	139
O8—H38⋯O6	0.82	1.84	2.570 (7)	148

**Fig. 1.**
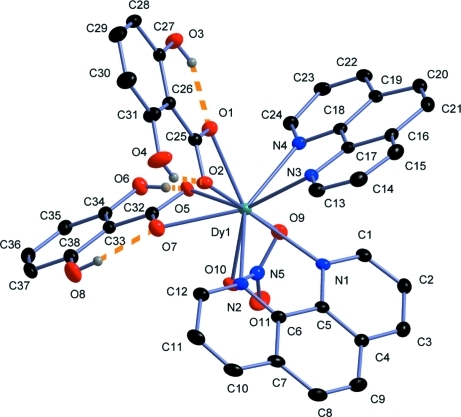
The molecular structure of the title compound. Displacement ellipsoids are drawn at the 30% probablility level. H atoms have been omitted for clarity. Dashed orange lines denote intramolecular hydrogen bonds.

## supplementary crystallographic information

### Comment

The description of the structure of the title compound is part of a series of papers on mononuclear complexes of the type [Ln(DHB)_2_(NO_3_)(phen)_2_] [Ln = Ce, Pr, Sm, Eu and Dy (this publication); DHB = 2,6-dihydroxybenzoate; phen = 1,10-phenanthroline]. All five compounds are isostructural to the previously reported Nd complex (Zheng *et al.* 2010).

### Experimental

All reagents are commercially available and of analytical grade. Dy(NO_3_)_3_.6H_2_O (0.229 g, 0.5 mmol), 2,6-dihydroxybenzoic acid (0.074 g, 0.5 mmol), 1,10-phenanthroline (0.090 g, 0.5 mmol) and NaHCO_3_ (0.042 g, 0.5 mmol) were dissolved in a water–ethanol solution (10 ml, v/v 1:1). The solution was refluxed for 4 h and filtered after cooling to room temperature. Yellow single crystals were obtained from the filtrate in 4 days.

### Refinement

H atoms were positioned geometrically and refined as riding atoms, with C—H = 0.93 and O—H = 0.82 Å and *U*_iso_(H) = 1.2*U*_eq_(C) and 1.5*U*_eq_(O). The highest residual electron density was found 0.90 Å from Dy1 and the deepest hole 1.61 Å from O1.

### Crystal data

**Table:**

[Dy(C_7_H_5_O_4_)_2_(NO_3_)(C_12_H_8_N_2_)_2_]	*F*(000) = 1772
*M**_r_* = 891.14	*D*_x_ = 1.751 Mg m^−^^3^
Monoclinic, *P*2_1_/*c*	Cu *K*α radiation, λ = 1.54184 Å
Hall symbol: -P 2ybc	Cell parameters from 5391 reflections
*a* = 11.1268 (3) Å	θ = 3.3–67.5°
*b* = 26.7587 (4) Å	µ = 12.46 mm^−^^1^
*c* = 14.2703 (5) Å	*T* = 298 K
β = 127.280 (2)°	Prism, yellow
*V* = 3380.72 (18) Å^3^	0.35 × 0.32 × 0.30 mm
*Z* = 4	

### Data collection

**Table:**

Oxford Diffraction Gemini S Ultra CCD diffractometer	5923 independent reflections
Radiation source: Enhance (Cu) X-ray Source	4674 reflections with *I* > 2σ(*I*)
graphite	*R*_int_ = 0.043
Detector resolution: 15.9149 pixels mm^-1^	θ_max_ = 67.1°, θ_min_ = 3.3°
ω scans	*h* = −11→13
Absorption correction: multi-scan (*CrysAlis PRO*; Oxford Diffraction, 2006)	*k* = −31→31
*T*_min_ = 0.097, *T*_max_ = 0.118	*l* = −16→17
11595 measured reflections	

### Refinement

**Table:**

Refinement on *F*^2^	Primary atom site location: structure-invariant direct methods
Least-squares matrix: full	Secondary atom site location: difference Fourier map
*R*[*F*^2^ > 2σ(*F*^2^)] = 0.051	Hydrogen site location: inferred from neighbouring sites
*wR*(*F*^2^) = 0.137	H-atom parameters constrained
*S* = 1.00	*w* = 1/[σ^2^(*F*_o_^2^) + (0.0774*P*)^2^] where *P* = (*F*_o_^2^ + 2*F*_c_^2^)/3
5923 reflections	(Δ/σ)_max_ < 0.001
496 parameters	Δρ_max_ = 1.68 e Å^−^^3^
0 restraints	Δρ_min_ = −1.16 e Å^−^^3^

### Fractional atomic coordinates and isotropic or equivalent isotropic displacement parameters (Å^2^)

**Table:**

	*x*	*y*	*z*	*U*_iso_*/*U*_eq_	
Dy1	0.92866 (4)	0.862990 (11)	0.22227 (3)	0.03846 (13)	
O1	0.8450 (5)	0.83886 (15)	0.0261 (4)	0.0522 (11)	
O2	0.8884 (5)	0.91905 (15)	0.0604 (4)	0.0499 (10)	
O3	0.7499 (6)	0.80195 (16)	−0.1748 (4)	0.0702 (15)	
H27	0.7912	0.8015	−0.1039	0.105*	
O4	0.8352 (8)	0.97830 (17)	−0.1043 (5)	0.087 (2)	
H31	0.8578	0.9702	−0.0400	0.131*	
O5	0.6975 (5)	0.81369 (15)	0.1368 (4)	0.0505 (10)	
O6	0.6691 (5)	0.89410 (16)	0.1017 (4)	0.0584 (12)	
O7	0.4806 (6)	0.76073 (17)	0.1052 (4)	0.0624 (12)	
H34	0.5706	0.7671	0.1414	0.094*	
O8	0.4205 (7)	0.93749 (19)	0.0292 (6)	0.0822 (17)	
H38	0.5048	0.9344	0.0461	0.123*	
O9	0.9472 (5)	0.80198 (15)	0.3727 (4)	0.0583 (12)	
O10	0.8436 (6)	0.87351 (16)	0.3467 (4)	0.0556 (11)	
O11	0.8566 (7)	0.8210 (3)	0.4675 (5)	0.0883 (19)	
N1	1.1603 (6)	0.87915 (17)	0.4391 (4)	0.0408 (11)	
N2	0.9734 (6)	0.95380 (16)	0.2914 (4)	0.0442 (12)	
N3	1.1761 (6)	0.86887 (16)	0.2491 (4)	0.0406 (11)	
N4	1.0506 (6)	0.78088 (18)	0.2394 (4)	0.0435 (11)	
N5	0.8816 (6)	0.8312 (2)	0.3985 (5)	0.0527 (13)	
C1	1.2533 (8)	0.8434 (2)	0.5122 (6)	0.0483 (14)	
H1	1.2385	0.8112	0.4820	0.058*	
C2	1.3706 (8)	0.8506 (3)	0.6301 (6)	0.0560 (16)	
H2	1.4331	0.8242	0.6768	0.067*	
C3	1.3928 (8)	0.8974 (3)	0.6766 (6)	0.0569 (17)	
H3	1.4692	0.9030	0.7561	0.068*	
C4	1.2998 (7)	0.9369 (2)	0.6037 (5)	0.0463 (14)	
C5	1.1836 (7)	0.9259 (2)	0.4851 (5)	0.0400 (12)	
C6	1.0847 (7)	0.9650 (2)	0.4065 (5)	0.0416 (13)	
C7	1.1054 (8)	1.0142 (2)	0.4516 (6)	0.0478 (15)	
C8	1.2255 (8)	1.0234 (2)	0.5743 (7)	0.0583 (18)	
H8	1.2388	1.0555	0.6044	0.070*	
C9	1.3175 (9)	0.9870 (3)	0.6454 (7)	0.0618 (19)	
H9	1.3950	0.9943	0.7238	0.074*	
C10	1.0063 (9)	1.0512 (2)	0.3732 (6)	0.0554 (17)	
H10	1.0169	1.0839	0.3991	0.066*	
C11	0.8959 (9)	1.0395 (2)	0.2603 (7)	0.0590 (18)	
H11	0.8283	1.0638	0.2082	0.071*	
C12	0.8835 (8)	0.9907 (2)	0.2220 (6)	0.0521 (15)	
H12	0.8078	0.9836	0.1432	0.063*	
C13	1.2359 (8)	0.9120 (2)	0.2500 (6)	0.0528 (15)	
H13	1.1765	0.9406	0.2248	0.063*	
C14	1.3846 (8)	0.9163 (3)	0.2873 (6)	0.0563 (17)	
H14	1.4228	0.9473	0.2875	0.068*	
C15	1.4723 (8)	0.8747 (3)	0.3231 (6)	0.0594 (18)	
H15	1.5715	0.8772	0.3488	0.071*	
C16	1.4132 (7)	0.8282 (3)	0.3211 (5)	0.0489 (15)	
C17	1.2625 (7)	0.8270 (2)	0.2827 (5)	0.0429 (13)	
C18	1.1934 (7)	0.7804 (2)	0.2724 (5)	0.0413 (13)	
C19	1.2765 (7)	0.7358 (2)	0.2975 (5)	0.0475 (14)	
C20	1.4291 (8)	0.7381 (3)	0.3411 (6)	0.0588 (18)	
H20	1.4846	0.7087	0.3620	0.071*	
C21	1.4956 (8)	0.7820 (3)	0.3529 (6)	0.0595 (18)	
H21	1.5963	0.7826	0.3820	0.071*	
C22	1.1985 (8)	0.6906 (2)	0.2765 (6)	0.0545 (17)	
H22	1.2462	0.6603	0.2879	0.065*	
C23	1.0545 (8)	0.6914 (2)	0.2399 (6)	0.0535 (17)	
H23	1.0027	0.6617	0.2261	0.064*	
C24	0.9842 (8)	0.7374 (2)	0.2232 (5)	0.0491 (15)	
H24	0.8857	0.7373	0.1994	0.059*	
C25	0.8441 (7)	0.8824 (2)	−0.0106 (5)	0.0425 (13)	
C26	0.7959 (7)	0.8895 (2)	−0.1304 (5)	0.0416 (13)	
C27	0.7498 (7)	0.8487 (2)	−0.2084 (5)	0.0482 (15)	
C28	0.7019 (8)	0.8562 (3)	−0.3224 (6)	0.0608 (19)	
H28	0.6718	0.8292	−0.3732	0.073*	
C29	0.6992 (9)	0.9035 (3)	−0.3599 (6)	0.069 (2)	
H29	0.6661	0.9082	−0.4367	0.083*	
C30	0.7440 (10)	0.9440 (3)	−0.2870 (6)	0.068 (2)	
H30	0.7418	0.9758	−0.3144	0.082*	
C31	0.7926 (9)	0.9375 (2)	−0.1728 (6)	0.0582 (18)	
C32	0.6131 (7)	0.8522 (2)	0.1013 (5)	0.0474 (15)	
C33	0.4611 (7)	0.8492 (2)	0.0671 (5)	0.0460 (14)	
C34	0.4014 (7)	0.8037 (3)	0.0719 (5)	0.0504 (15)	
C35	0.2580 (7)	0.8010 (3)	0.0423 (5)	0.0555 (17)	
H35	0.2199	0.7707	0.0459	0.067*	
C36	0.1724 (8)	0.8441 (3)	0.0071 (6)	0.067 (2)	
H36	0.0754	0.8422	−0.0139	0.080*	
C37	0.2255 (9)	0.8898 (3)	0.0021 (6)	0.067 (2)	
H37	0.1658	0.9183	−0.0214	0.081*	
C38	0.3692 (8)	0.8923 (3)	0.0329 (6)	0.0594 (18)	

### Atomic displacement parameters (Å^2^)

**Table:**

	*U*^11^	*U*^22^	*U*^33^	*U*^12^	*U*^13^	*U*^23^
Dy1	0.0455 (2)	0.02877 (18)	0.0461 (2)	−0.00037 (13)	0.03041 (17)	−0.00158 (13)
O1	0.065 (3)	0.039 (2)	0.050 (2)	−0.0029 (19)	0.033 (2)	0.0047 (18)
O2	0.066 (3)	0.040 (2)	0.050 (2)	−0.0004 (19)	0.038 (2)	−0.0041 (18)
O3	0.083 (4)	0.044 (2)	0.057 (3)	−0.004 (2)	0.028 (3)	−0.008 (2)
O4	0.154 (6)	0.043 (2)	0.066 (3)	−0.019 (3)	0.067 (4)	−0.005 (2)
O5	0.046 (2)	0.041 (2)	0.064 (3)	0.0088 (18)	0.033 (2)	0.0041 (19)
O6	0.060 (3)	0.044 (2)	0.075 (3)	0.002 (2)	0.043 (3)	0.009 (2)
O7	0.059 (3)	0.052 (2)	0.075 (3)	0.002 (2)	0.041 (3)	0.010 (2)
O8	0.076 (4)	0.053 (3)	0.110 (4)	0.019 (3)	0.053 (3)	0.017 (3)
O9	0.066 (3)	0.040 (2)	0.069 (3)	0.000 (2)	0.040 (3)	0.000 (2)
O10	0.067 (3)	0.049 (2)	0.070 (3)	0.004 (2)	0.052 (3)	−0.002 (2)
O11	0.097 (4)	0.129 (5)	0.066 (3)	−0.013 (4)	0.063 (3)	0.010 (3)
N1	0.053 (3)	0.033 (2)	0.046 (3)	0.002 (2)	0.035 (2)	0.0000 (19)
N2	0.063 (3)	0.029 (2)	0.046 (3)	0.003 (2)	0.035 (3)	0.0012 (19)
N3	0.043 (3)	0.037 (2)	0.050 (3)	−0.006 (2)	0.033 (2)	−0.004 (2)
N4	0.051 (3)	0.038 (2)	0.043 (2)	−0.005 (2)	0.029 (2)	−0.001 (2)
N5	0.050 (3)	0.060 (3)	0.052 (3)	−0.006 (3)	0.033 (3)	−0.005 (3)
C1	0.061 (4)	0.041 (3)	0.055 (4)	0.006 (3)	0.042 (3)	0.004 (3)
C2	0.053 (4)	0.063 (4)	0.051 (4)	0.009 (3)	0.031 (3)	0.007 (3)
C3	0.047 (3)	0.072 (4)	0.051 (3)	−0.006 (3)	0.029 (3)	−0.006 (3)
C4	0.043 (3)	0.049 (3)	0.053 (3)	−0.007 (3)	0.032 (3)	−0.008 (3)
C5	0.045 (3)	0.039 (3)	0.049 (3)	−0.005 (2)	0.035 (3)	−0.005 (2)
C6	0.052 (3)	0.033 (3)	0.053 (3)	−0.006 (2)	0.039 (3)	−0.003 (2)
C7	0.064 (4)	0.036 (3)	0.072 (4)	−0.015 (3)	0.056 (4)	−0.013 (3)
C8	0.074 (5)	0.044 (3)	0.083 (5)	−0.021 (3)	0.061 (4)	−0.022 (3)
C9	0.063 (4)	0.062 (4)	0.068 (4)	−0.023 (4)	0.043 (4)	−0.026 (4)
C10	0.086 (5)	0.029 (3)	0.081 (5)	−0.004 (3)	0.067 (4)	−0.003 (3)
C11	0.084 (5)	0.034 (3)	0.079 (5)	0.012 (3)	0.060 (4)	0.014 (3)
C12	0.068 (4)	0.037 (3)	0.059 (4)	0.004 (3)	0.042 (3)	0.002 (3)
C13	0.061 (4)	0.045 (3)	0.060 (4)	−0.005 (3)	0.041 (3)	−0.005 (3)
C14	0.059 (4)	0.060 (4)	0.064 (4)	−0.023 (3)	0.044 (4)	−0.012 (3)
C15	0.053 (4)	0.077 (5)	0.055 (4)	−0.019 (4)	0.036 (3)	−0.010 (3)
C16	0.041 (3)	0.067 (4)	0.040 (3)	−0.006 (3)	0.026 (3)	−0.006 (3)
C17	0.045 (3)	0.046 (3)	0.038 (3)	−0.003 (3)	0.026 (3)	−0.007 (2)
C18	0.048 (3)	0.045 (3)	0.034 (3)	0.000 (3)	0.027 (3)	−0.002 (2)
C19	0.056 (4)	0.051 (3)	0.040 (3)	0.012 (3)	0.032 (3)	0.004 (3)
C20	0.059 (4)	0.064 (4)	0.049 (4)	0.016 (3)	0.030 (3)	0.005 (3)
C21	0.047 (4)	0.084 (5)	0.050 (4)	0.007 (4)	0.031 (3)	0.000 (3)
C22	0.073 (5)	0.039 (3)	0.052 (4)	0.012 (3)	0.039 (3)	0.002 (3)
C23	0.073 (5)	0.032 (3)	0.055 (4)	0.000 (3)	0.039 (3)	−0.003 (3)
C24	0.051 (3)	0.038 (3)	0.056 (4)	−0.009 (3)	0.031 (3)	−0.006 (3)
C25	0.045 (3)	0.041 (3)	0.046 (3)	−0.001 (2)	0.030 (3)	−0.001 (2)
C26	0.044 (3)	0.040 (3)	0.041 (3)	−0.002 (2)	0.026 (3)	−0.001 (2)
C27	0.040 (3)	0.048 (3)	0.047 (3)	0.005 (3)	0.021 (3)	−0.005 (3)
C28	0.055 (4)	0.072 (5)	0.049 (4)	0.000 (3)	0.028 (3)	−0.017 (3)
C29	0.078 (5)	0.084 (5)	0.048 (4)	−0.010 (4)	0.039 (4)	−0.005 (4)
C30	0.094 (6)	0.063 (4)	0.059 (4)	−0.010 (4)	0.052 (4)	0.007 (3)
C31	0.078 (5)	0.047 (3)	0.052 (4)	−0.010 (3)	0.040 (4)	0.000 (3)
C32	0.050 (4)	0.049 (3)	0.040 (3)	0.003 (3)	0.026 (3)	0.002 (3)
C33	0.041 (3)	0.058 (3)	0.040 (3)	0.004 (3)	0.025 (3)	−0.002 (3)
C34	0.045 (3)	0.067 (4)	0.042 (3)	0.001 (3)	0.028 (3)	0.000 (3)
C35	0.047 (4)	0.079 (5)	0.044 (3)	−0.002 (3)	0.029 (3)	0.000 (3)
C36	0.048 (4)	0.105 (6)	0.049 (4)	0.007 (4)	0.031 (3)	−0.001 (4)
C37	0.057 (4)	0.085 (5)	0.060 (4)	0.029 (4)	0.035 (4)	0.008 (4)
C38	0.058 (4)	0.066 (4)	0.053 (4)	0.015 (3)	0.033 (3)	0.006 (3)

### Geometric parameters (Å, °)

**Table:**

Dy1—N1	2.597 (5)	C9—H9	0.9300
Dy1—N2	2.556 (4)	C10—C11	1.344 (10)
Dy1—N3	2.545 (5)	C10—H10	0.9300
Dy1—N4	2.515 (5)	C11—C12	1.389 (9)
Dy1—O1	2.442 (4)	C11—H11	0.9300
Dy1—O2	2.557 (4)	C12—H12	0.9300
Dy1—O5	2.458 (4)	C13—C14	1.403 (9)
Dy1—O6	2.444 (5)	C13—H13	0.9300
Dy1—O9	2.604 (5)	C14—C15	1.360 (11)
Dy1—O10	2.482 (5)	C14—H14	0.9300
O1—C25	1.274 (7)	C15—C16	1.401 (10)
O2—C25	1.275 (7)	C15—H15	0.9300
O3—C27	1.340 (8)	C16—C17	1.414 (8)
O3—H27	0.8196	C16—C21	1.439 (10)
O4—C31	1.345 (8)	C17—C18	1.425 (8)
O4—H31	0.8201	C18—C19	1.417 (8)
O5—C32	1.275 (7)	C19—C22	1.410 (10)
O6—C32	1.281 (8)	C19—C20	1.413 (10)
O7—C34	1.348 (8)	C20—C21	1.344 (11)
O7—H34	0.8198	C20—H20	0.9300
O8—C38	1.352 (9)	C21—H21	0.9300
O8—H38	0.8190	C22—C23	1.352 (10)
O9—N5	1.267 (7)	C22—H22	0.9300
O10—N5	1.277 (7)	C23—C24	1.398 (9)
O11—N5	1.205 (7)	C23—H23	0.9300
N1—C1	1.330 (8)	C24—H24	0.9300
N1—C5	1.363 (7)	C25—C26	1.462 (8)
N2—C12	1.326 (8)	C26—C31	1.411 (9)
N2—C6	1.363 (8)	C26—C27	1.415 (8)
N3—C13	1.328 (8)	C27—C28	1.386 (10)
N3—C17	1.360 (8)	C28—C29	1.365 (11)
N4—C24	1.322 (8)	C28—H28	0.9300
N4—C18	1.356 (8)	C29—C30	1.371 (11)
C1—C2	1.382 (9)	C29—H29	0.9300
C1—H1	0.9300	C30—C31	1.383 (10)
C2—C3	1.367 (10)	C30—H30	0.9300
C2—H2	0.9300	C32—C33	1.452 (9)
C3—C4	1.402 (10)	C33—C34	1.407 (9)
C3—H3	0.9300	C33—C38	1.418 (9)
C4—C5	1.404 (8)	C34—C35	1.383 (9)
C4—C9	1.433 (9)	C35—C36	1.384 (11)
C5—C6	1.439 (8)	C35—H35	0.9300
C6—C7	1.420 (8)	C36—C37	1.379 (12)
C7—C10	1.398 (9)	C36—H36	0.9300
C7—C8	1.442 (10)	C37—C38	1.380 (10)
C8—C9	1.329 (11)	C37—H37	0.9300
C8—H8	0.9300		
			
O1—Dy1—O6	79.39 (17)	C9—C8—H8	119.4
O1—Dy1—O5	74.42 (15)	C7—C8—H8	119.4
O6—Dy1—O5	53.02 (13)	C8—C9—C4	121.4 (6)
O1—Dy1—O10	143.53 (17)	C8—C9—H9	119.3
O6—Dy1—O10	70.42 (17)	C4—C9—H9	119.3
O5—Dy1—O10	71.23 (16)	C11—C10—C7	120.0 (6)
O1—Dy1—N4	71.90 (15)	C11—C10—H10	120.0
O6—Dy1—N4	134.73 (16)	C7—C10—H10	120.0
O5—Dy1—N4	85.41 (15)	C10—C11—C12	119.4 (6)
O10—Dy1—N4	116.50 (15)	C10—C11—H11	120.3
O1—Dy1—N3	79.34 (16)	C12—C11—H11	120.3
O6—Dy1—N3	142.59 (16)	N2—C12—C11	123.8 (6)
O5—Dy1—N3	145.01 (14)	N2—C12—H12	118.1
O10—Dy1—N3	137.05 (16)	C11—C12—H12	118.1
N4—Dy1—N3	64.45 (15)	N3—C13—C14	122.9 (6)
O1—Dy1—N2	122.95 (14)	N3—C13—H13	118.5
O6—Dy1—N2	79.54 (16)	C14—C13—H13	118.5
O5—Dy1—N2	127.03 (16)	C15—C14—C13	119.2 (6)
O10—Dy1—N2	71.66 (16)	C15—C14—H14	120.4
N4—Dy1—N2	145.66 (16)	C13—C14—H14	120.4
N3—Dy1—N2	86.76 (16)	C14—C15—C16	119.9 (7)
O1—Dy1—O2	51.87 (12)	C14—C15—H15	120.0
O6—Dy1—O2	71.26 (16)	C16—C15—H15	120.0
O5—Dy1—O2	107.97 (14)	C15—C16—C17	117.4 (6)
O10—Dy1—O2	130.24 (14)	C15—C16—C21	123.7 (7)
N4—Dy1—O2	112.92 (15)	C17—C16—C21	118.9 (6)
N3—Dy1—O2	71.39 (15)	N3—C17—C16	122.5 (6)
N2—Dy1—O2	71.20 (14)	N3—C17—C18	117.6 (5)
O1—Dy1—N1	145.48 (16)	C16—C17—C18	119.9 (6)
O6—Dy1—N1	132.75 (16)	N4—C18—C19	122.9 (6)
O5—Dy1—N1	131.74 (15)	N4—C18—C17	118.2 (5)
O10—Dy1—N1	70.26 (16)	C19—C18—C17	118.8 (6)
N4—Dy1—N1	86.66 (15)	C22—C19—C20	123.5 (6)
N3—Dy1—N1	66.91 (16)	C22—C19—C18	116.4 (6)
N2—Dy1—N1	63.96 (15)	C20—C19—C18	120.1 (6)
O2—Dy1—N1	118.90 (14)	C21—C20—C19	121.2 (6)
O1—Dy1—O9	124.35 (14)	C21—C20—H20	119.4
O6—Dy1—O9	105.28 (16)	C19—C20—H20	119.4
O5—Dy1—O9	67.00 (15)	C20—C21—C16	120.9 (7)
O10—Dy1—O9	49.88 (15)	C20—C21—H21	119.5
N4—Dy1—O9	66.62 (16)	C16—C21—H21	119.5
N3—Dy1—O9	112.12 (16)	C23—C22—C19	120.2 (6)
N2—Dy1—O9	112.21 (15)	C23—C22—H22	119.9
O2—Dy1—O9	174.91 (14)	C19—C22—H22	119.9
N1—Dy1—O9	66.19 (15)	C22—C23—C24	119.2 (6)
C25—O1—Dy1	97.6 (4)	C22—C23—H23	120.4
C25—O2—Dy1	92.2 (3)	C24—C23—H23	120.4
C27—O3—H27	109.5	N4—C24—C23	123.4 (7)
C31—O4—H31	109.4	N4—C24—H24	118.3
C32—O5—Dy1	93.5 (4)	C23—C24—H24	118.3
C32—O6—Dy1	94.0 (4)	O1—C25—O2	118.3 (5)
C34—O7—H34	109.5	O1—C25—C26	120.4 (5)
C38—O8—H38	109.5	O2—C25—C26	121.3 (5)
N5—O9—Dy1	94.6 (3)	C31—C26—C27	117.5 (6)
N5—O10—Dy1	100.3 (4)	C31—C26—C25	121.1 (5)
C1—N1—C5	116.9 (5)	C27—C26—C25	121.4 (5)
C1—N1—Dy1	123.6 (4)	O3—C27—C28	118.1 (6)
C5—N1—Dy1	119.2 (4)	O3—C27—C26	121.2 (6)
C12—N2—C6	117.3 (5)	C28—C27—C26	120.7 (6)
C12—N2—Dy1	122.6 (4)	C29—C28—C27	119.7 (6)
C6—N2—Dy1	119.8 (3)	C29—C28—H28	120.2
C13—N3—C17	118.1 (6)	C27—C28—H28	120.2
C13—N3—Dy1	123.0 (4)	C28—C29—C30	121.6 (7)
C17—N3—Dy1	118.1 (4)	C28—C29—H29	119.2
C24—N4—C18	117.7 (5)	C30—C29—H29	119.2
C24—N4—Dy1	122.8 (4)	C29—C30—C31	119.8 (7)
C18—N4—Dy1	119.4 (4)	C29—C30—H30	120.1
O11—N5—O9	123.8 (6)	C31—C30—H30	120.1
O11—N5—O10	121.0 (6)	O4—C31—C30	117.9 (6)
O9—N5—O10	115.2 (5)	O4—C31—C26	121.4 (6)
N1—C1—C2	124.5 (6)	C30—C31—C26	120.7 (6)
N1—C1—H1	117.8	O5—C32—O6	117.8 (6)
C2—C1—H1	117.8	O5—C32—C33	121.1 (6)
C3—C2—C1	118.6 (6)	O6—C32—C33	121.0 (6)
C3—C2—H2	120.7	C34—C33—C38	117.3 (6)
C1—C2—H2	120.7	C34—C33—C32	121.3 (6)
C2—C3—C4	119.6 (6)	C38—C33—C32	121.3 (6)
C2—C3—H3	120.2	O7—C34—C35	116.8 (6)
C4—C3—H3	120.2	O7—C34—C33	121.8 (6)
C3—C4—C5	117.7 (6)	C35—C34—C33	121.4 (6)
C3—C4—C9	123.0 (6)	C34—C35—C36	118.7 (7)
C5—C4—C9	119.4 (6)	C34—C35—H35	120.6
N1—C5—C4	122.7 (5)	C36—C35—H35	120.6
N1—C5—C6	117.4 (5)	C37—C36—C35	122.4 (7)
C4—C5—C6	119.9 (5)	C37—C36—H36	118.8
N2—C6—C7	122.0 (5)	C35—C36—H36	118.8
N2—C6—C5	119.1 (5)	C36—C37—C38	118.5 (7)
C7—C6—C5	118.9 (5)	C36—C37—H37	120.8
C10—C7—C6	117.5 (6)	C38—C37—H37	120.8
C10—C7—C8	123.5 (6)	O8—C38—C37	117.8 (7)
C6—C7—C8	119.1 (6)	O8—C38—C33	120.6 (7)
C9—C8—C7	121.3 (6)	C37—C38—C33	121.7 (7)

### Hydrogen-bond geometry (Å, °)

**Table:**

*D*—H···*A*	*D*—H	H···*A*	*D*···*A*	*D*—H···*A*
O3—H27···O1	0.82	1.86	2.577 (7)	146
O4—H31···O2	0.82	1.86	2.588 (6)	148
O7—H34···O5	0.82	1.91	2.592 (7)	139
O8—H38···O6	0.82	1.84	2.570 (7)	148
